# Clinical trial design for cutaneous neurofibromas

**DOI:** 10.1212/WNL.0000000000005790

**Published:** 2018-07-10

**Authors:** Ashley Cannon, Kurt Jarnagin, Bruce Korf, Brigitte C. Widemann, Denise Casey, Hon-Sum Ko, Jaishri O. Blakeley, Sharad K. Verma, Dominique C. Pichard

**Affiliations:** From the Department of Genetics (A.C., B.K.), University of Alabama at Birmingham; BioPharm Tech (K.J.), San Mateo, CA; Pediatric Oncology Branch (B.C.W.) and Dermatology Branch, Center for Cancer Research (D.C.P.), National Cancer Institute, NIH, Bethesda; Division of Oncology Products (D.C.) and Division of Dermatology and Dental Products (H.-S.K.), Food and Drug Administration, Silver Spring; and Department of Neurology (J.O.B., S.K.V.), The Neurofibromatosis Therapeutic Acceleration Program, The Johns Hopkins University School of Medicine, Baltimore, MD.

## Abstract

**Objective:**

Several clinical trials targeting cutaneous neurofibromas (cNF) have been conducted; however, none has resulted in meaningful changes to care. The Clinical Trial Design and Development subgroup's goals were to (1) define key considerations in the design of clinical trials for cNF, (2) summarize existing data in relation to these considerations, and (3) provide consensus recommendations about key elements of trial design to accelerate the clinical development of therapies for cNF.

**Methods:**

The subgroup, with experts from genetics, dermatology, neurology, oncology, and basic science, spanning academia, government research, and regulatory programs, and industry, reviewed published and unpublished data on clinical trials for cNF and other diseases in the skin. Discussions of these data resulted in formulation of a list of priority issues to address in order to develop efficient and effective clinical trials for cNF.

**Results:**

The subgroup identified 2 natural history studies of cNF, 4 priority outcome measures, and 6 patient-reported outcome tools for potential use in efficacy trials of cNF. Time to initiate intervention, patient eligibility, mechanism of action, route of administration, safety monitoring, and regulatory agency interactions were identified as key factors to consider when designing clinical trials for cNF.

**Conclusions:**

Alignment on endpoints and methods for the measurement and quantification of cNF represent a priority for therapeutic development for cNF. Advances in technological methods and outcome tools utilized in other skin diseases may be applicable to cNF studies. Patient age is an important factor guiding trial design and clinical development path.

Cutaneous neurofibromas (cNF) are histologically benign tumors. Although these tumors are not lethal, they are associated with substantial morbidity due to disfigurement and severe effects on quality of life (QoL) for persons with neurofibromatosis 1 (NF1). A QoL survey completed by 128 adults with NF1 in France demonstrated that visibility of lesions negatively influenced emotions, physical symptoms, and functioning.^1^ Similar results were reported in Italian and US cohorts.^2,3^ These findings underscore the need for effective therapy and prevention. Other than procedural-based approaches, there are no drug therapies that have successfully altered the occurrence, progression, or size of cNF lesions.^4^ Given the severe morbidity of cNF in patients with NF1, the NF1 research community and NF1 advocacy groups have placed a high priority on developing therapies to reduce the burden of cNF. The Clinical Trial Design and Development subgroup presents the priorities and challenges associated with conducting clinical trials targeting cNF in NF1 patients. Our aspiration is that this information may enable clinical investigators, scientists, regulatory agencies, affected individuals, and their advocates to be better positioned to work together to accelerate the development of therapies for cNF.

## Methods

The Clinical Trial Design and Development subgroup was composed of clinical and clinical science experts from academic, public agency, and private sector settings tasked with the review of published and unpublished materials pertaining to the conduct of clinical trials for cNF and other diseases in the skin (e.g., atopic dermatitis, psoriasis). Key topic areas including natural history, assessment methods for measuring tumors, functional endpoints, safety, regulatory interactions, and development strategies were discussed. The subgroup members reviewed the topic areas individually and as a group during a series of meetings facilitated over a 4-month period in order to prioritize key questions and establish consensus recommendations for each topic area.

## Results

### Natural history

The Food and Drug Administration rare disease guidance emphasizes the importance of defining the natural history of a rare disorder in order to guide clinical trial design and the development of therapies.^5^ Knowledge of disease course and characteristics is critical for selecting outcome measures and other important design elements in clinical trials. Unique challenges exist in the study of the natural history of NF1, including the small number of patients and the heterogeneous clinical phenotype. Most data on the natural history of cNF in patients with NF1 are based on retrospective studies. Recent efforts are addressing this gap and 2 prospective natural history studies on cutaneous manifestations of NF1 are ongoing. The first of these 2 studies measured the number and size of cNF lesions with calipers in 3 different body sites of 22 adults (median age 47.5, range 38–70) over 8 years.^6^ Clinical assessments were performed at 4-month intervals for the first 2 years and then at 8 years from study entry. The data revealed an average monthly increase in volume of 0.37 mm^3^ for tumors located in the back region, 0.28 mm^3^ in the abdominal region, and 0.21 mm^3^ in the arm/leg region (1.7%–2.7% volume increase per month).^6^ The mean number of new cNF lesions within the 100 cm2 study frames by body region developing over 8 years was 3.1 on the back, 1.7 on the abdomen, and 0.4 on the extremities. Involution was not observed. These data show that cNF lesions grow very slowly in adults older than 35 years, and that the rate of increase in number of cNF lesions varies by body region.

**Figure 1:**
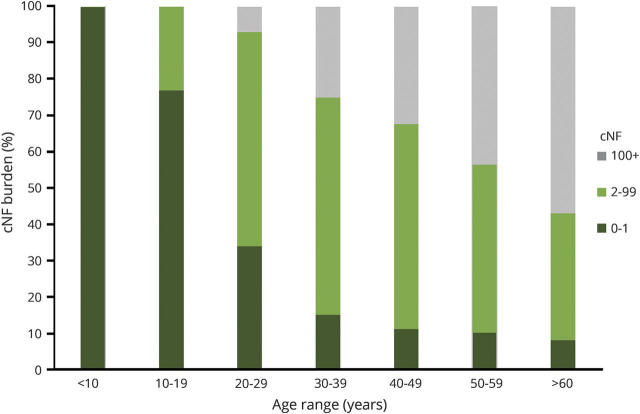
Cutaneous neurofibroma (cNF) burden increase by age Adapted with permission from the Duong et al.^8^ cross-sectional study of 728 pediatric and adult patients with neurofibromatosis 1.

The second prospective study is the National Cancer Institute's natural history study of children and adults up to the age of 35 with NF1 (NCT00924196). In this study, the presence and number of skin manifestations related to NF1 (café-au-lait macules, cNF lesions, axillary/inguinal freckling) are monitored every 1–3 years. Data are still being collected. In addition to monitoring growth over time across the overall population of individuals with NF1, quantifying growth characteristics at different ages were identified as a key consideration for further defining the natural history of cNF. A population-based study conducted in Wales demonstrated that over 99% of adults with NF1 had cNF lesions and that the number of lesions increased with age.^7^ A subsequent cross-sectional study of 728 pediatric and adult patients with NF1 also showed that the number of cNF lesions increased with age (figure 1).^8^ In addition to age, hormones and pregnancy have been hypothesized to influence the development of cNF.^9^

These studies provide a broad review of growth characteristics of cNF in children and adults with NF1. However, knowledge gaps remain, and they include (1) prospective data on the rate of appearance of various forms of cNF development and pattern of progression in children and young adults, (2) growth rates of nascent vs mature cNF tumors, (3) the influence of hormones or other growth factors on the development and proliferation of cNF tumors, (4) the rate of spontaneous involution, (5) identification of reliable biomarkers for cNF development, and (6) comparisons of measurement tools. Inconsistent definitions that have been applied to cNF pose a major limitation in generating natural history data.^10^ It will be important to define consistent criteria for cNF (and possible subtypes) a priori and to clarify which tumor types should be measured with specific assessment tools within each study.

### Clinical trials and outcome measures

Outcome measures are determined by the intended goals of the clinical trial. The cost, ease of use, accessibility, and reliability and reproducibility of the outcome measure are of paramount importance. Possible efficacy endpoints for trials in cNF are shown in table 1, and include assessment of (1) tumor size, (2) number of tumors, and (3) patient QoL.

**Table 1:**
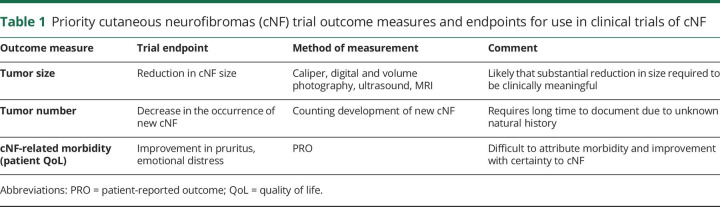
Priority cutaneous neurofibromas (cNF) trial outcome measures and endpoints for use in clinical trials of cNF

### Assessment of tumor number and size

**Figure 2:**
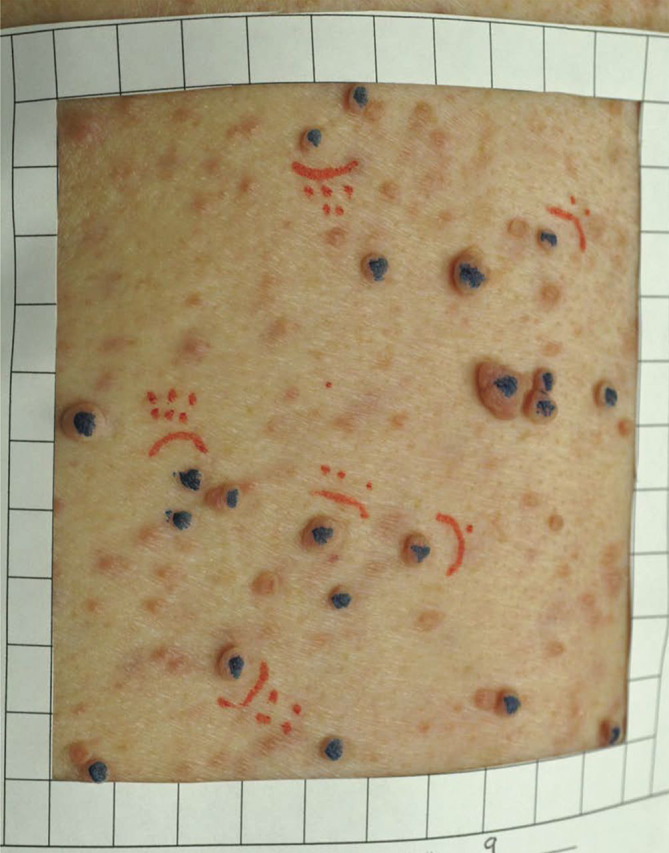
100 cm^2^ paper frame used as a guide to manually count and measure cutaneous neurofibroma (cNF) lesions Blue indicates cNF lesions that were counted (>4 mm). Red indicates the cNF lesions that were measured.

Measurement approaches that enable the collection of quantitative data about tumor growth are very important. The paper frame is a clinician-reported outcome measure used to quantify the number of cNF lesions (figure 2).^11^ A 100 cm^2^ paper frame is placed on the skin and all cNF lesions within the frame are counted. A caliper is used to measure lesion size. While the use of calipers in the evaluation of cNF size after treatment with the vascular endothelial growth factor–targeting agent ranibizumab resulted in variable results,^4^ caliper measurement was used effectively with good reliability for the evaluation of cNF in an 8-year natural history study.^6^ The combination of calipers and paper frames represents a low-cost, accessible method to report the number and the size of cNF lesions in a limited area of the body. Disadvantages are the inability to detect or measure small lesions, the need for investigator experience and training, and the impracticality of measuring the whole body tumor burden when thousands of tumors are present. Initial data indicate that this approach captures change in tumor size, but cannot assess the size of small or flat tumors or portions beneath the skin surface.

New approaches, such as optical coherence tomography (OCT) or high-frequency ultrasound (HFUS), may assess the full thickness of a lesion above and below the skin surface. These procedures may allow a more sensitive measure of change over time or in response to therapy.^12,13^ The high resolution of these techniques may shorten the observation period and provide the opportunity to intervene earlier in the course of cNF development. OCT can provide a visualization of the full structure of normal human skin at a resolution of approximately 5 μm, enabling the visualization of sweat ducts, capillaries, and blood flow. However, nerve endings are under 2 μm in diameter, which is below the resolution of OCT, so there are limitations with respect to visualization of some aspects of the cNF.

HFUS is a painless, noninvasive technique that uses frequencies between 20 and 100 MHz to provide increased resolution and improved visualization of structures and inflammation in the skin. HFUS has been used to measure tumors in the skin, such as basal cell carcinomas, and to aid in the surgical removal of basal cell carcinomas.^14^ A recent prospective study with the aims of (1) describing sonographic appearance of different types of cNF, (2) assessing interobserver agreement, and (3) associating clinical and ultrasound features evaluated 108 cNF lesions in patients with NF1 with a 25-MHz HFUS.^13^ This study demonstrated consistent sonographic findings, including tumors being hypoechoic. The identification of a specific sonographic feature may avoid the need for a skin biopsy. This avoidance would be especially useful in children, where early diagnosis is more challenging. Additional studies need to be performed to validate the use of HFUS in this population.

**Figure 3:**
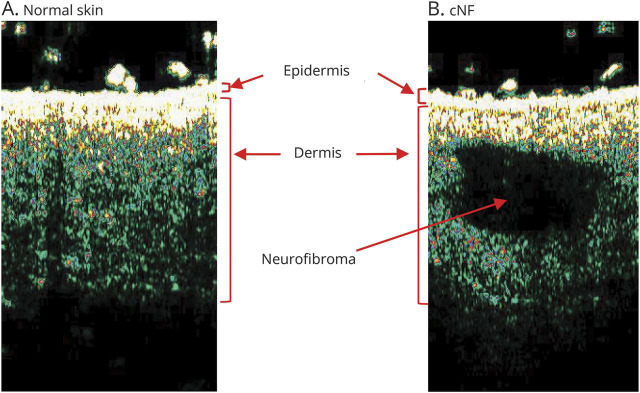
High-frequency ultrasound of skin Comparison of normal skin (A) to skin with cutaneous neurofibromas (cNF) (B) using a 20-MHz transducer on the DermaScan C ultrasound. The neurofibroma is hypoechoic.

Technological advances can address some shortcomings of existing quantification methods and other outcome measures that have been proposed. They include 2D or 3D volumetric photography, conventional ultrasound, and MRI. Conventional ultrasound and HFUS have been used to quantify the volume of individual cNF lesions (figure 3), but the inability to capture whole body burden remains.^13,15^ To address this limitation, a whole body imaging system that uses multiple cameras to simultaneously capture the entire body surface for 2D and 3D image analysis has been developed, but has not been applied to cNF to date. While tools such as conventional ultrasound, MRI, OCT, and HFUS offer the potential for rapid and enhanced sensitivity of quantifying tumor size and measuring tumor growth on an accelerated time scale, the cost and training required to use these devices need to be considered. Clinical trials in rare diseases often require participation of multiple sites, and costly devices or substantial training requirements may pose barriers for such trials.

Clinically meaningful outcomes may not necessarily be captured with the assessment of tumor size or number alone. Complete resolution of cNF or dramatic reduction in size may be representative of clinical benefit for patients; however, more modest partial reductions in tumor burden have less clear meaningful clinical benefit. In general, tumor size reduction that correlates with reduced pain or discomfort or improved function would provide more supportive evidence of clinically meaningful effectiveness.

In addition to the assessment tools to objectively measure change in cNF size, an ordinal global assessment scale, such as the Investigator Global Assessment or the Physician Global Assessment, should be considered for the study of cNF in NF1. Properly designed global assessment scores are acceptable as outcome measures for establishing primary endpoints in clinical trials for some dermatologic conditions,^16^ such as atopic dermatitis, psoriasis, and acne. Global assessments have also been used in studies for infantile hemangioma,^17^ which is also not life-threatening, but does result in disfigurement and functional deficits, and has a negative influence on the patient's psyche. Such global assessment scales should be based on a limited number of levels describing the skin condition under study, and not on changes from an arbitrary time point such as baseline. Differences between global assessment scales for a specific condition underscore the need for standardization among investigators within the field such that data may be compared across studies.^16^ Currently, there is not a global assessment scale for cNF, and thus development of such a scoring system is an unmet need for clinical research in this area.

### Effect on patient well-being

Patient-reported outcomes (PROs) are likely to be important in the assessment of therapeutic efficacy for cNF to provide information regarding the patients' perceived burden of disease, benefit of an intervention, or burden of drug toxicity. There are currently no validated PRO instruments for cNF. However, 2 PRO instruments have been studied extensively in patients with cutaneous manifestations of NF1: Short Form (SF)–36 and Skindex.^1–3^ SF-36 is a QoL survey to assess general physical and emotional health. Skindex is an instrument to measure the effect of skin disease on QoL, and has been validated for several dermatologic conditions including psoriasis and atopic dermatitis. Although studies evaluating cNF Skindex have consistently demonstrated that visibility of cNF lesions is associated with negative effects on QoL, dedicated validation for its use in studies in NF1-associated cNF is lacking.

There are other PRO measures for various dermatologic conditions based on disease-related symptoms, emotions, or function (table 2).^18–23^ One may consider adapting them for use in cNF trials. For instance, pruritus is an important symptom in patients with NF1 and has been shown to reduce QoL^24^; however, pruritus in NF1 may not be specific to cNF lesions. One study reported that pruritus was localized to sites of cNF in only 52% of the patients evaluated.^25^ In addition to a validated PRO measure in the NF1 population, the subgroup acknowledged the importance of availability of the instrument in multiple languages and cross-cultural relevance of its components.

**Table 2:**
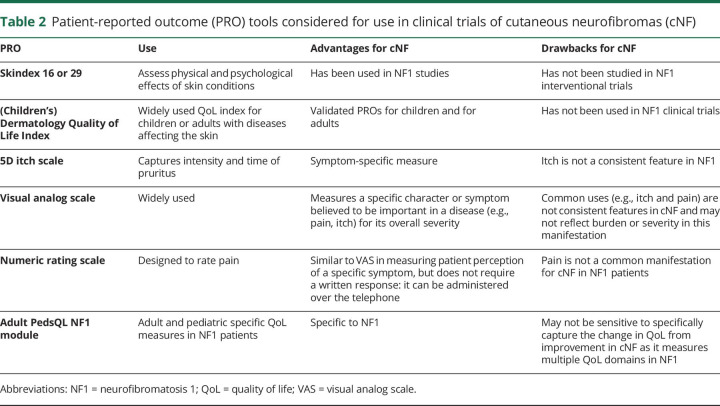
Patient-reported outcome (PRO) tools considered for use in clinical trials of cutaneous neurofibromas (cNF)

### Biologic endpoints

Tissue and blood biomarkers may be used as surrogates of tumor growth and might provide valuable endpoints for clinical trials. Thus far, there has been a concerted effort to identify biomarkers in plexiform neurofibromas, though progress has been limited. If pruritus could serve as a potential disease activity indicator, analytes relating to pruritus (e.g., enzymes from mast cell degranulation) might hold potential as biologic markers. However, such markers as those relating to pruritus may not be specific for cNF tumor growth, as discussed above.

### Important intervention considerations

There are numerous considerations for interventions targeting cNF in NF1. Key factors to consider when designing clinical trials on cNF include the following:

1. Time to initiate the intervention: We do not have adequate information on the biology of the cNF of individual patients to confidently address this question. Despite that, a pediatric study plan should be considered early in clinical development of drugs for cNF.
2. Eligibility for the intervention: Clinical trials should ensure diversity in enrollment with respect to age, sex, race, and geographic location, and stratify as biologically appropriate for the drug.
3. Mechanism of action of the intervention: Evaluation of mechanism of action may entail inclusion of biopsy at baseline and after the intended therapeutic window, where the collected tissue can be used to determine the effect of the intervention at the pharmacologic or molecular level.
4. Route of administration of the intervention: Risk–benefit considerations favor the use of directed therapies (topical or intralesional) when feasible as these approaches limit systemic toxicity.^4^
5. Safety monitoring for administering the intervention: Clinical studies should have appropriate safety monitoring according to what is known about the intervention (e.g., from nonclinical and available clinical data) and the intended use (as treatment or prevention) so as to plan for the level of toxicity for discontinuation of intervention, dose adjustment, and rescue measures.
6. Regulatory considerations and interactions with regulatory agencies: US regulations require demonstration of both safety and substantial evidence of effectiveness under the conditions of use prescribed, recommended, or suggested in labeling.^26^ Demonstration that the potential benefits of the treatment outweigh the risks is necessary. Evidence of effectiveness is based on well-controlled studies of adequate design to show a significant effect on a clinically meaningful endpoint in the targeted population. For cNF, tumor shrinkage or response rate alone may not be sufficient evidence of clinical benefit. Although many oncology products have been granted accelerated approval on the basis of radiologic response rate as a surrogate endpoint that is reasonably likely to predict clinical benefit (i.e., a survival effect), the clinical relevance of achieving a partial response in a benign tumor is less certain. Objective response rates of sufficient magnitude and durability, according to a well-defined response definition specific to cNF lesions, may be considered as a primary endpoint, and incorporation of a suitable PRO, either as a co-primary or key secondary endpoint, could provide evidence of clinical benefit to patients.

## Discussion

The NF1 field is at an inflection point; the level of understanding of the pathogenesis of tumors, the cells of origin, and the influence of the cellular environment has advanced in recent years. Continued progress in new technologies has provided new tools to identify and measure change in cNF as objective outcome measures. As scientists continue to learn more about the biology of cNF, therapies directed at the prevention or ablation of cNF tumors in NF1 may be developed. The success of any development program for intervention on cNF in patients with NF1 will be dependent on the early and frequent interactions among regulators, NF experts, and patient groups to design and conduct appropriate trials. As noted in the opening article in this series,^27^ the Response Evaluation in Neurofibromatosis & Schwannomatosis International Collaboration (ccrod.cancer.gov/confluence/display/REINS/Cutaneous+Neurofibromas) has recently formed the cNF Working Group as one step in this process. In addition, therapeutic approaches for cNF could potentially utilize benefits and incentives provided to the orphan disease community.^4^ Continued collaborative approaches across the broad research community will support efficient and effective clinical trial design for NF1-associated cNF lesions.

## Glossary

cNF: cutaneous neurofibromas
HFUS: high-frequency ultrasound
NF1: neurofibromatosis type 1
OCT: optical coherence tomography
PRO: patient-reported outcomes
QoL: quality of life
SF: Short Form
